# An Episcopal Surgeon

**Published:** 1894-12-01

**Authors:** 


### An Episcopal Surgeon.

Few departments of medical history so are interesting, or have been so little investigated, as that which deals with the relations of medicine to the Church during the early Middle Ages. The influence of Christianity upon medicine has been highly complex, and has varied for good or evil according as its charitable, ascetic, or mystical tendencies predominated. Owing to a natural reaction against the licentiousness of heathenism, charity, the chief virtue of the New Testament, was early replaced by asceticism, love of one's neighbour giving way to hatred of one's own body. It was the Christian's first duty to keep under his body. In this contest disease and debility were to be looked upon as allies rather than enemies, and if resisted at all were certainly not to be resisted by earthly means. So strongly are these views expressed by the foremost theologians of the second century that there seemed some danger lest medicine should be authoritatively declared an unchristian profession, though Christ had Himself asserted that the sick need physicians, and though a large part of the New Testament had been written by a medical practitioner. It seemed to Marcion (105—165) incredible that a man who made it his business to tend the vile body should have been so highly favoured, and he boldly declared that Col. iv. 14 was spurious. Marcion fell into heresy, and failed to expunge "the beloved physician" from our Bibles, but it was, perhaps, something of the same spirit which enabled an unfounded sixth century legend to overshadow the assertion of an Apostle and to convert the medical evangelist into a painter. Tatian (110—180), the famous author of the Diatessaron, went still further. He declared that medicine is unchristian, that drugs were created by the devil, and are themselves inhabited by demons, who enter therewith into men's bodies and draw them away from their trust in God.

Similar tendencies may be traced in later periods. When the priest alone could read medical books, charity demanded that he should use his knowledge to compound potions for his sick neighbours, but the mystic asceticism above noticed forbade him to use hands sanctified by the service of the altar for the painful and often repulsive operations of surgery, whence partly the disastrous separation between the two divisions of the healing art. It is not intended to discuss here so vast a subject as the influence of theology on medicine, but the above remarks may serve as preface to the following short history, which is interesting both as the earliest record of a successful abdominal section and as illustrating some of the points referred to. The story is translated from cap. iv. of the "Lives of the Bishops of Merida," written by Paul the Deacon, A.D. 610.

"How Paul, a Greek physician, became Bishop of Merida (570), and how he delivered a woman from great peril of childbirth. Paul came from the East to Merida, and having lived there a long while and distinguished himself by many virtues, especially kindliness and humility, God caused him to be chosen bishop, and afterwards granted to his prayers a remission of those tumults which disturbed the Church in the days of his predecessor.

"Now it happened that the wife of a noble citizen of senatorial rank conceived a child, the which was crushed in her womb (*in ventre collisus est*). And though many physicians tried many things she got no better, but came in great peril, and was in daily expectation of death. So her illustrious husband, to whom nothing was dearer than his wife, dismissing all the doctors, hastened in hope of salvation to the holy bishop, and falling at his feet, besought him with tears that, inasmuch as he was a servant of God he would pray for his wife, and inasmuch as he was a physician he would deign himself to put a hand to her cure. But the man of God answered, 'It is not lawful for me to do as you wish, seeing I am an unworthy priest of the Lord, and often sacrifice with my hands to the Lord, wherefore I cannot do what you say, lest afterwards I place polluted hands on the holy altar, and incur the wrath of the divine Majesty.' And he added, 'Let us go, in God's name, and visit her, and I will send the church physicians (*medicos ecclesiæ*), and will instruct them as I am able, but I cannot do ought with my own hands.' But he, knowing that the others were useless, and that his wife was nearly dead, wept sore, and entreated him to do what he could himself. And when the bishop would in no wise consent, all the brethren present besought him with tears to go. But he said, 'I know God is very merciful, and I trust that, should I heal this woman, He would at once pardon my presumption, but I doubt not that evil-minded persons would hereafter cast the same in my teeth.' Then all the brethren exclaimed, 'Not one of us will speak a word of it, but go, sir, and do with all speed what you can.' At last he promised to go, but lest by doing ought rashly he should afterwards hardly find place for repentance, he went first to the church of the most blessed Virgin Eulalia, and lay prostrate in prayer on the pavement all that day and the following night. Then, having received the Divine sanction, he went at once to the sick woman's house, and having prayed, laid hands on her in the name of the Lord. Hoping in God, he made with great skill a very fine incision with a fine knife, and extracted the already putrid infant limb by limb." (Extra-uterine pregnancy?)

To condense the sequel, the patient perfectly recovered, and they then made an estimate of their wealth, "which was so great that no senator in the Province of Lusitania was richer than they," and offered half to the bishop, who, after many refusals, accepted it, "not for himself but for the poor." Shortly after both died, "and the rest of their wealth came to the most holy Bishop Paul, who, having entered Merida a penniless stranger, became richer than all the nobles, for the whole possessions of the Church might be considered as nothing compared with his wealth."

